# Placental bands on MRI in the setting of placenta accreta spectrum: Case report with radiologic-pathologic correlation

**DOI:** 10.1016/j.radcr.2022.10.086

**Published:** 2022-11-24

**Authors:** Robert Weinstein, Arthur Vaught, Alexander Baras, Erin Gomez

**Affiliations:** a Johns Hopkins School of Medicine, 733 North Broadway, Baltimore, MD 21205, USA; b Division of Maternal Fetal Medicine, Department of Gynecology and Obstetrics, Johns Hopkins University School of Medicine, Baltimore, MD 21205, USA; c Department of Pathology, Johns Hopkins University School of Medicine, Baltimore, MD 21205, USA; d The Russell H. Morgan Department of Radiology and Radiological Science, Johns Hopkins University School of Medicine, Baltimore, MD 21205, USA

**Keywords:** Placental bands, Placenta accreta, MRI, Pathology

## Abstract

Placental bands on T2-weighted magnetic resonance imaging (MRI) are a known imaging finding in placenta accreta spectrum (PAS). It is believed that these linear T2 hypo-intensities may reflect increased fibrin deposition in the setting of placental hemorrhage or infarct. However, to date there is little published data regarding histopathologic analysis of placental parenchyma at the site of identified bands. We report the case of a 34-year-old female with a single placental band demonstrated on preoperative MRI which was evaluated postoperatively and found to represent a placental infarct.

## Introduction

MRI is an important tool in the diagnosis of placenta accreta spectrum (PAS), with key imaging features consisting of heterogeneous placental signal intensity, placental bands on T2-weighted imaging, abnormal placental contour and/or bulging, and direct visualization of myometrial invasion [[1], [2]–3]. To date, the clinical significance and etiology of placental bands is unknown, but has been hypothesized to represent areas of fibrous tissue due to increased fibrin seen in previous histopathology in cases of placental invasion [4,5]. Other potential causes of these bands include aberrant placental vasculature, which has been found in pathological specimens of confirmed PAS [6]. These alterations in the normal vasculature of the placenta can result in microvascular infarctions, eventually leading to the fibrin deposition seen on imaging. T2 dark flow voids may mimic placental bands on MRI and/or confound identification.

## Case presentation

**Fig. 1:**
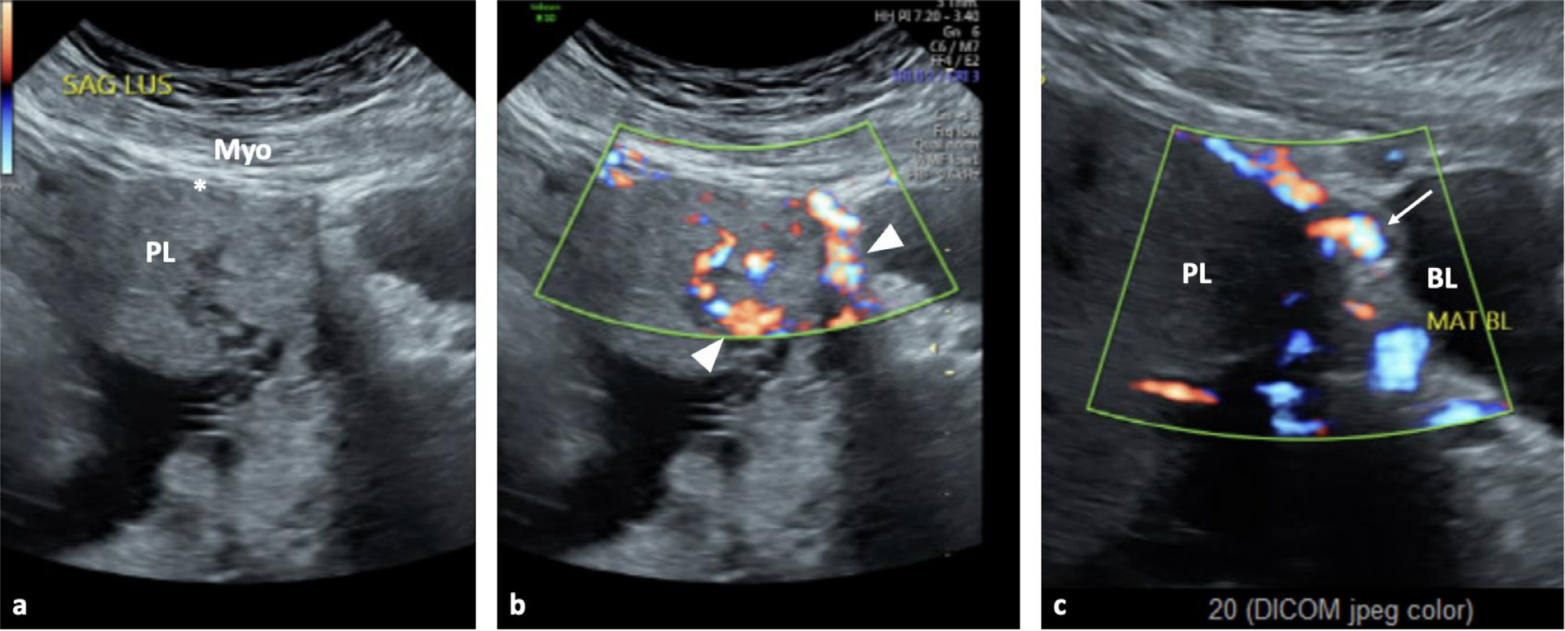
Grayscale (A) and color Doppler (B) transabdominal ultrasound images at the level of the lower uterine segment demonstrating loss of the retroplacental hypoechoic space (asterisk) and vascular lacunae (arrowhead) with placenta (PL) directly abutting the myometrium (Myo). Additional color Doppler ultrasound image of the pelvis (C) demonstrates increased vascularity with probable uterine wall invasion (arrows) adjacent to the bladder (BL).

A 34-year-old woman, G4P3003, presented at 30-weeks’ gestation for consultation regarding high concern for PAS on prenatal ultrasound. She had a history of one prior cesarean section and 2 prior vaginal deliveries. This was not an IVF pregnancy, and she had no history of additional uterine surgeries or instrumentation. Ultrasound demonstrated placenta previa with multiple placental lacunae, loss of the normal retroplacental hypoechoic space, and increased vascularity with uterine wall invasion noted by color Doppler (Fig. 1), highly concerning for placenta percreta [7,8].

**Fig. 2:**
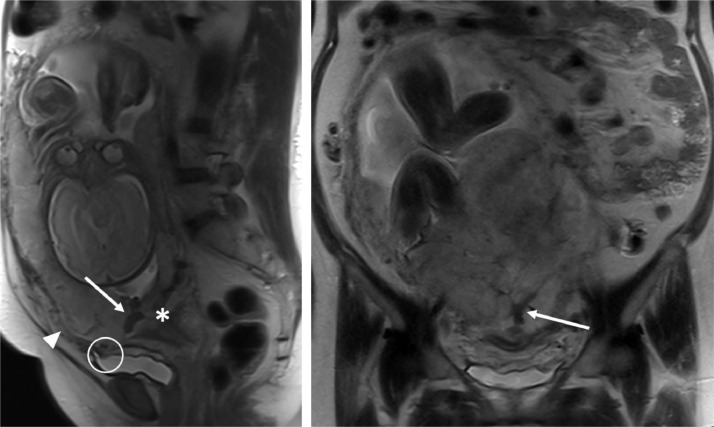
Sagittal (A) and coronal (B) T2-weighted images of the abdomen and pelvis demonstrating findings concerning for placenta accreta spectrum, including myometrial thinning and placental bulging in the lower uterine segment (arrowhead). A thick, T2-hypointense placental band is present (arrows). Complete placenta previa (asterisk) and focal interruption of the bladder wall (circle) are also noted.

To further characterize the placenta, non-contrast MRI of the abdomen and pelvis was performed. MRI findings included complete placenta previa, lobulated placental contour, and a prominent T2 hypointense band in the anteroinferior placenta (Fig. 2), all concerning for PAS. Focal discontinuity of the myometrium near the bladder dome was also noted, with irregularity and abnormal T2 signal within the bladder wall in this region, raising concern for placenta percreta with focal invasion of the urinary bladder.

A plan was made to deliver the fetus at 34-weeks’ gestation via cesarean hysterectomy. Bilateral uterine artery embolization was performed by interventional radiology intraoperatively after the cesarean section and before the hysterectomy. Intraoperative findings were consistent with placenta percreta with placenta visualized protruding through to the uterine serosa in the left lower uterine segment with confirmation of bladder invasion. The case had an estimated blood loss of 4500 mL, and she received 5 units of packed red blood cell, 3 units of fresh frozen plasma, and 2 units of cryoprecipitate. Although there was placental invasion into the detrusor, there was no invasion of the bladder mucosa and cystotomy was avoided. The patient progressed well and was discharged home on postoperative day 5. There were no neonatal complications outside of iatrogenic prematurity and both mother and baby were doing well at 6-week postpartum exam.

**Fig. 3:**
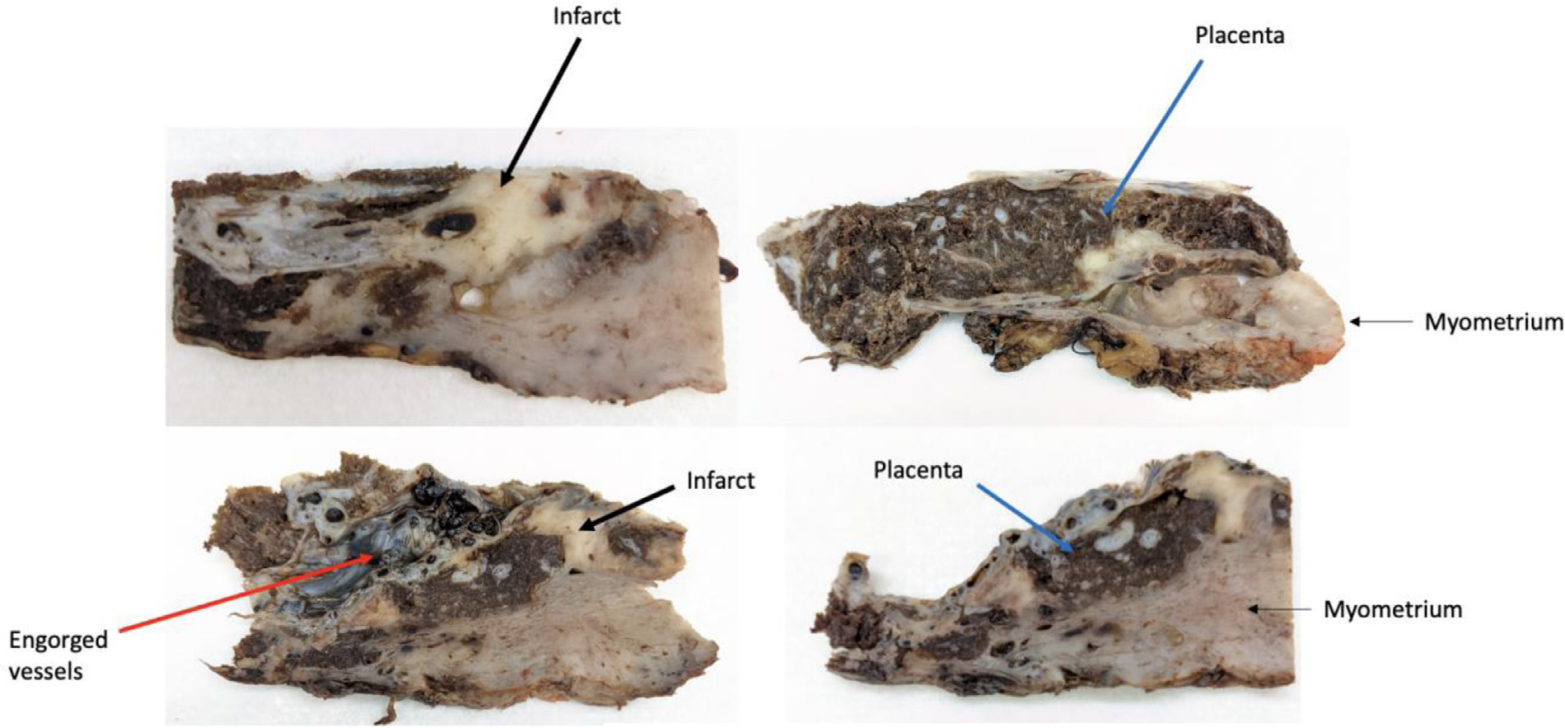
Gross pathologic specimens of the placenta (blue arrow). Focal band of white tissue in the lower left portion of the specimen (thick arrow) are most consistent with infarct-associated fibrosis, corresponding to the region of the placental band noted on MR imaging. Engorged vessels (red arrow) are seen on the middle of the specimen, a finding consistent with PAS. Normal myometrium (thin arrows) is seen subjacent to the placenta.

Hysterectomy specimens including the morbidly adherent placenta were sent to pathology following the procedure for formal evaluation of the myometrial-placental interface, as well as the site of the placental band noted on MRI. The placental parenchyma was noted to be dark red brown with focal tan-white discoloration, measuring up to 2 cm. Areas of fibrosis concerning for infarct were noted and can be seen in Fig. 3.

## Discussion

PAS is typically diagnosed in the second or third trimester of pregnancy via ultrasound imaging, with MRI serving as an adjunctive tool in diagnosis and surgical planning [9]. Placental bands on T2-weighted MR imaging are a known imaging feature of PAS. An increased understanding of the etiology and clinical significance of this finding may aid physicians in the management of these patients. In this case, the patient had a singular placental band that could be easily identified in the anterior margin of the placenta near the internal cervical os. This anatomic landmark allowed the specimen to be more easily evaluated at pathology, where it was noted to be a region of dense fibrous tissue most consistent with placental infarction. Placental infarction has been previously correlated with disease severity in patients with preeclampsia [10]. Additional studies have also found significant associations between increased rates of abnormal placentation and the presence of placental infarcts [11].

The current standard management for patients with PAS is a planned fundal cesarean hysterectomy at 32-24 weeks, with the surgical approach influenced by the degree of vascular recruitment or structural invasion [12]. Current systematic reviews have found the sensitivity and specificity of MRI to be 94.4% and 84.0%, respectively [13]. However, there is little evidence correlating findings with the severity of symptoms experienced by PAS patients, including blood loss or surgical complications. Improved imaging methods and understanding of radiologic findings may provide increased insight into the pathophysiology of this condition and help guide physicians in the clinical management of this patient population.

## Conclusion

In summary, this case describes a patient with complete placenta previa and suspected PAS who presented for cesarean hysterectomy. MR imaging demonstrated features of placenta accreta spectrum including the presence of a singular placental band on T2-weighted images, which was found to represent a dense region of fibrous tissue consistent with placental infarct at pathological evaluation. This finding can help build upon the current hypothesis that placental bands represent areas of fibrous tissue due to increased fibrin deposition. Abnormal placentation has been previously associated with placental infarction, which could occur following pathologic development of the uteroplacental vasculature [11]. The infarcted tissue could subsequentially undergo fibrosis on recovery leading to the deposition of fibrin seen on this and other pathology specimens over time. Further knowledge in this area may influence further research into placental bands as a predictor of clinical outcomes in PAS.

## Patient consent

The patient reported in the manuscript signed the informed consent/authorization for participation in research which includes the permission to use data collected in future research projects including presented case details and images used in this manuscript.
